# Resistance to treatment in eating disorders: A critical challenge

**DOI:** 10.1186/1471-244X-13-294

**Published:** 2013-11-07

**Authors:** Giovanni Abbate-Daga, Federico Amianto, Nadia Delsedime, Carlotta De-Bacco, Secondo Fassino

**Affiliations:** 1 Eating Disorders Center for Treatment and Research, Department of Neuroscience, University of Turin, Turin, Italy

**Keywords:** Anorexia nervosa, Eating disorders, Psychodynamic psychotherapy, Resistance, Change, Insight, Therapeutic relationship, Maintaining factors, Defense mechanisms, Countertransference

## Abstract

**Background:**

Current literature on Eating Disorders (EDs) is devoid of evidence-based findings providing support to effective treatments, mostly for anorexia nervosa (AN). This lack of successful guidelines may play a role in making these disorders even more resistant. In fact, many individuals do not respond to the available treatments and develop an enduring and disabling illness. With this overview we aimed to highlight and discuss treatment resistance in AN – with an in-depth investigation of resistance-related psychological factors.

A literature search was conducted on PubMed and PsychINFO; English-language articles published between 1990 and 2013 investigating the phenomenon of resistance to treatment in AN have been considered.

**Discussion:**

The selected papers have been then grouped into four main thematic areas: denial of illness; motivation to change; maintaining factors and treatment outcome; and therapeutic relationship. Eating symptomatology was found to only partially explain resistance to treatment. The role of duration of illness has been questioned whilst some maintaining factors seemed promising in providing a useful framework for this phenomenon. Emotive and relational aspects have been investigated on their role in resistance as well as therapists’ countertransference.

**Summary:**

Remarkably there has been little research done on resistance to treatment in the ED field, in spite of its clinical relevance. Motivation, insight and subjective meaning of the illness can be useful tools to manage the resistance phenomenon when coupled with a wider approach. The latter enables the therapists to be aware of their role in the therapeutic alliance through countertransference aspects and to consider the EDs as disorders of the development of both personality and self, entailing severe impairments as regards identity and relationships.

## Background

Resistance to treatment has been variously interpreted across psychiatric disorders with this concept being often used as a synonym of difficult-to-treat disorders. In literature, there is a general consensus that the available treatments for major psychiatric disorders frequently result in either a lack of effect or a moderate response. Accordingly, to challenge treatment-resistance is fundamental in psychiatric clinical practice and it also represents a crucial public health problem [1].

Two issues should be carefully addressed while studying the phenomenon of treatment resistance. First, patients’ apparent strong wish for treatment often not resulting in any improvement, and second, the profound resistance – with conscious and unconscious aspects – related to the distress triggered by changing [2] which is a hallmark of Eating Disorders (EDs).

EDs are serious and complex mental illnesses [3] with a biopsychosocial pathogenesis [4] and that are often characterized by a chronic and disabling course [5]. Recent follow-up studies failed to provide evidence that treatments can influence long-term results [6] with relevant consequences on public health-related costs [7-9]. The only exception is represented by adolescents affected by anorexia nervosa (AN) reporting a more favorable outcome when compared to adults [10]. In fact, one-third of adult AN patients show poor outcome also because of an enduring and refractory illness [5,11] whilst many maintaining factors of the AN symptomatology are less entrenched in younger patients [10].

In AN, both avoidance of treatment [12] and dropouts - ranging between 20 and 51% in the inpatient population and from 23 to 73% in outpatient samples – are related to the great difficulty existing in engaging and maintaining their treatment adherence [13,14].

In AN, willingness to improve represents only one of several factors required to achieve recovery. In fact, reluctance to recover is sustained by ego-syntonic symptoms that reinforce the illness [15-17] and relapse is common [18,19] also because patients often perceive consequences of AN as positive and adaptive [20]. Symptoms and pathologic beliefs are indeed intertwined in AN, revealing a self-protecting meaning. Eating psychopathology is underpinned by several entangled biopsychosocial elements - personality, general psychopathology, environment, and treatment itself - that may have a role in both increasing and lowering patients’ willingness to recover.

For many years, the possible meanings of eating symptomatology have been debated; studies investigating patients’ perspectives have finally demonstrated how patients often describe AN as a means of obtaining identity, avoiding negative emotions, and satisfying a strong need for control [21]. Patients are reluctant and ambivalent about changing and they can unconsciously oppose a strong resistance to their therapists’ efforts towards improvement. Defense mechanisms that protect patients by negative feelings are often directly activated by therapy paving the way to resistance [2]. The peculiar defense mechanisms in AN – denial and intellectualization – can become resistance to treatment when patients are asked to face both changes and negative affect [22,23]. AN should be considered indeed not only as a constellation of physical and psychological symptoms but also as an array of self-protecting behaviors able to give patients respite from distress [24].

Although these aspects are well-known to clinicians, there is a dearth of organized and critical studies in the AN field on resistance to treatment and motivational therapies.

The aim of the present paper is to gather and discuss the studies published from 1990 to 2013 on the topic of treatment resistance in AN since a better understanding of poor motivation to treatment is fundamental to provide more tailored therapies. The research questions underpinning this article arose from the need of a deeper understanding of 4 core areas with regard to AN treatment: namely, we considered the role of: 1. illness awareness; 2. patients’ willingness; 3. psychopathological factors ED symptomatology-related; 4. therapist.

## Methods

Article search has been conducted using PubMed and PsychINFO databases using the following MeSH terms: “anorexia nervosa”, “bulimia nervosa”, “eating disorders”, “motivational change”, “insight”, “predictors of outcome”, “treatment resistance”, “decision making”, “psychosis”, “countertransference”. The first three terms have been alternately used as fixed research criteria. This MeSH search yielded 142 articles pertaining to treatment resistance in EDs.

Abstracts or full texts have been then gathered and selected by two different researchers (ND and AF) to check the following inclusion criteria: a) AN diagnosis; b) original research or debate articles, c) being published after 1990, and d) English language. Exclusion criteria were: a) samples diagnosed with unstandardized instruments or according to unspecified criteria; b) case reports; c) letters; and d) editorials.

We decided to include not only original research but also debate articles for two major reasons. First, to date the relationship with patients has been scarcely investigated, mostly by qualitative studies and debate-framed articles. Second, currently there is such a dearth of evidence-based data in the treatment of AN that the opinion of highly experienced researchers and clinicians is extremely valuable.

According to the aforementioned criteria we finally selected 71 articles to be included in this overview whilst 71 have been excluded because not meeting the inclusion criteria.

We could not perform a meta-analysis because of the differences we found among studies regarding sample composition, methodology used in the assessment of patients, and different outcome criteria. To achieve greater clarity, the gathered articles have been divided into four thematic areas grounded on the 4 aforementioned research questions (1. role of illness awareness; 2. role of patients’ willingness; 3. role of psychopathological factors ED symptomatology-related; 4. therapist’s role): denial versus insight of illness; motivation to change; maintaining factors and treatment outcome; therapeutic relationship, countertransference, management of treatment resistance (see Table 1). Studies encompassing more than one area have been distributed under more sections.

**Table 1:** Articles included in the clinical overview; they have been divided into 4 thematic areas based on 4 research questions: 1. role of illness awareness; 2. role of patients’ willingness; 3. role of psychopathological factors 4. therapist’s role

**Thematic area**	**Number of articles**	**Authors**	**Number of patients**	**Diagnoses**	**Type of study**
Denial versus insight of illness	7	Couturier & Lock, [25]	86	AN	Retrospective cohort
		Gothelf et al., [26]	281	37 AN;163 other psychiatric disorders; 81 HC	Cross sectional
		Konstantakopoulos et al., [27]	75	25 AN; 15 BN; 35 HC	Cross sectional
		Konstantakopoulos et al., [28]	72	39 AN; 33 BN	Cross sectional
		Schoen et al., [29]	14	AN; BN (no subgroups)	Retrospective cohort
		Steinglass et al., [30]	25	AN	Cross sectional
		Viglione et al., [31]	38	AN	Cross sectional
Motivation to change	33	Ametller et al., [32]	70	AN	Prospective cohort
		Ben Tovim et al., [33]	220	95 AN; 88 BN; 37 EDNOS	Longitudinal cohort
		Bewell et al., [34]	127	AN	Prospective cohort
		Burket & Hodgin, [35]	72	49 BN; 11 ANBP; 6 EDNOS; 6 ANR	Retrospective cohort
		Carter et al., [36]	100	AN	Prospective cohort
		Casasnovas et al., [37]	218	58 AN; 95 BN; 65 EDNOS	Cross sectional
		Cavedini et al., [38]	38	AN	Prospective cohort
		Clausen, [39]	78	35 AN; 30 BN; 13 EDNOS	Prospective cohort
		Danner et al., [40]	16	AN	Cross sectional
		Darcy et al., [41]	20	AN	Cross sectional
		Gale et al., [42]	202	62 BN; 140 AN	Cross sectional
		Geller et al., [43]	99	15 ANBP ; 32 ANR ; 41 ANST; 7 BN; 4 BNST	Test validation
		Geller et al., [44]	63	24 ANR; 12 ANBP; 21 ANST; 7 BN	Prospective cohort
		Geller et al., [45]	128	10 ANR; 9 ANBP; 46 BN; 61 EDNOS	Longitudinal cohort
		Herzog et al., [46]	246	61 AN; 95 BN; 90 EDNOS	Prospective cohort
		Karlsson et al., [47]	89	AN	Prospective cohort
		Konstantakopoulos et al., [28]	72	39 AN; 33 BN	Cross sectional
		Lund et al., [48]	79	AN	Prospective cohort
		McHugh et al., [49]	65	AN	Prospective cohort
		Mewes et al., [50]	100	AN	Prospective cohort
		Muñoz et al., [51]	385	61 AN; 47 BN; 245 EDNOS	Prospective cohort
		Nordbø et al., [20]	18	AN	Cross sectional
		Nordbø et al., [52]	18	AN	Cross sectional
		Nordbø et al., [53]	36	AN	Cross sectional
		Ricca et al., [54]	103	53 AN; 50 ANST	Prospective cohort
		Rieger et al., [55]	44	AN	Test validation
		Salbach-Andrae et al., [56]	57	AN	Prospective cohort
		Schebendach et al., [57]	47	AN recovered	Prospective cohort
		Steinglass et al., [30]	25	AN	Cross sectional
		Touyz et al., [58]			Debate article
		Treasure et al., [59]			Debate article
		Wade et al., [60]	47	AN	Prospective cohort
		Ward et al., [61]	35	22 ANR; 11 ANBP; 2 BN	Cross sectional
Maintaining factors and treatment outcome	22	Castro et al., [62]	158	AN	Prospective cohort
		Clausen, [39]	78	35 AN; 30 BN; 13 EDNOS	Prospective cohort
		Danielsen & Rø, [63]	50	30 AN; 9 BN; 11 EDNOS	Prospective cohort
		Deter et al., [64]	81	AN	Prospective cohort
		Fairburn et al., [65]			Debate article
		Fassino et al., [66]	40	AN	Prospective cohort
		Fassino et al., [67]	42	AN	Prospective cohort
		Fassino et al., [68]	57	28 AN; 29 BN	Prospective cohort
		Federici & Kaplan, [69]	15	AN	Cross sectional
		Fichter et al., [18]	103	AN	Prospective cohort
		Geller et al., [70]			Debate article
		Goddard et al., [71]	74	63 AN; 7 BN; 4 EDNOS	Prospective cohort
		Helverskov et al., [72]	312	58 AN; 68 EDNOS-AN; 111 BN; 75 EDNOS-BN	Prospective cohort
		Hjern et al., [73]	748	AN	Prospective cohort
		Löwe et al., [74]	84	AN	Prospective cohort
		Salbach-Andrae et al., [56]	57	AN	Prospective cohort
		Schmidt & Treasure, [75]			Debate article
		Signorini et al., [76]	58	AN	Prospective cohort
		Steinhausen et al., [77]	60	48 ANR; 6 ANBP; 5 Atypical AN; 1 BN	Prospective cohort
		Sutandar-Pinnock et al., [78]	73	AN	Prospective cohort
		Treasure & Russell., [10]			Debate article
		Vrabel et al., [79]	74	13 AN; 37 BN; 24 EDNOS	Prospective cohort
Therapeutic relationship, countertransference, management of treatment resistance	18	Carter et al., [36]	100	AN	Prospective cohort
		Darcy et al., [41]	20	AN	Cross sectional
		Federici & Kaplan, [69]	15	AN	Cross sectional
		Feld et al., [80]	19	8ANR; 4ANBP; 4 BN; 3 EDNOS	Prospective cohort
		Forget et al., [81]			Debate article
		Geller et al., [82]	181	Cases: 3 ANBP; 4 ANR; 22 BN; 28 EDNOS	RCT
				Controls:3 ANBP; 4 ANR; 21 BN; 28 EDNOS	
		Gulliksen et al., [53]	38	AN	Cross sectional
		Karlsson et al., [47]	89	AN	Prospective cohort
		Masson & Sheeshka, [83]			Qualitative interview
		Moyers & Rollnick, [84]			Debate article
		Satir et al., [85]	120 (HP)		Cross sectional
		Skårderud et al., [24]			Debate article
		Strober et al., [86]			Debate article
		Tierney & Fox, [87]	53 (HP)		Cross sectional
		Tozzi et al., [88]	70	AN	Cross sectional
		Treasure et al., [59]			Debate article
		Vitousek et al., [89]			Debate article
		Warren et al., [90]	43 (HP)		Cross sectional

*AN* Anorexia Nervosa, *BN* Bulimia Nervosa, *ANR* Anorexia Nervosa Restricting subtype, *ANBP* Anorexia Nervosa Binge-Purging subtype, *EDNOS* Eating Disorder Not Otherwise Specified, *ANST* Subthreshold Anorexia Nervosa, *BNST* Subthreshold Bulimia Nervosa, *EDNOS-AN* anorexia-like eating disorder not otherwise specified-anorexia nervosa, *EDNOS-BN* anorexia-like eating disorder not otherwise specified-bulimia nervosa, *HC* Healthy Controls, *HP* healthcare practitioners, *RCT* randomized controlled trial.

## Results

### Sample composition

We included in this overview 71 studies with a variable sample composition ranging from 14 to 748 participants. No reviews or meta-analysis on this topic were available. Three of the considered studies recruited clinicians instead of patients [85,87,90] to investigate their countertransference. As regards the study design, we found debate articles, randomized controlled trial (RCT), qualitative interview, test validations, longitudinal cohort, cross-sectional, prospective cohort, and retrospective cohort studies (see Table 1).

### Diagnosis

As regards ED diagnosis, according to Diagnostic and Statistical Manual criteria (DSM-IV-TR) [91], 32 studies included only AN individuals, in one case recovered-AN; 9 papers considered bulimia nervosa (BN) and AN individuals, in one case compared to healthy controls (HC); 14 studies AN, BN and eating disorder not otherwise specified (EDNOS) participants. A study compared AN patients with subthreshold-AN individuals. In another work have been included patients with other psychiatric diagnoses and HC. The remaining studies were debate articles or did not consider affected individuals.

### Thematic areas

#### Denial versus insight of illness (7 studies)

Konstantakopoulos and Coworkers [27] highlighted how the restricting AN subtype is strongly characterized by lack of insight of illness and how the latter correlates with cognitive flexibility as measured with the trail making test. A couple of studies included in this area underscored that poor insight can assume delusional features, defining then a specific subgroup of AN patients even more treatment-resistant [28,30].

Moreover, two studies highlighted how insight of illness can be a helpful element in overcoming scarce compliance with therapy. Schoen and Coworkers [29] in a recent paper demonstrated a correlation between insight of illness and the seeking of professional treatments whilst other authors [31] supported indeed that duration of illness correlates with greater insight rather than with a more severe disorder.

Other three studies toned down the role of insight in overcoming poor compliance to treatments: it could be not only the lack of insight to maintain the disorder but also the combination of mature and immature defense mechanisms [26]. In fact, the majority of treatment-resistant patients show a clear denial of illness rather than scarce insight [27]. Moreover, it has been recently shown by the same authors – not in line with previous studies [31] – that insight is not related to duration of illness and that poor insight can be maintained also in a chronic phase of the ED [27]. Finally, Couturier and Lock [25] demonstrated how denial of illness does not impact significantly the outcome of family therapy.

#### Motivation to change (33 studies)

A few studies evaluated psychological determinants of motivation to change and only one paper investigated the correlation between motivation to change and quality of life as perceived by patients [51] but - given its cross-sectional design - it is not clear whether motivation can be influenced by quality of life or vice versa.

As regards cognitive factors, only one paper [38] found neurocognitive traits, in particular decision making impairment, as possibly involved in determining a less favorable outcome after Cognitive Behavioral Therapy (CBT), as well as lower weight gain and poorer motivation to change. AN patients would be cognitively oriented to choose short-term rather than long-term rewards with these features entailing poorer compliance to treatments and a less positive outcome. Recently, Danner and Coworkers [40] highlighted a significant correlation between set-shifting and central coherence raising the hypothesis of a relevant link – as regards prognosis – between these traits and treatment outcome. However, the authors underscored also that such deficits are shared by both ill and recovered-AN individuals so they cannot be predictors of motivation to change.

Eleven studies included in this thematic area investigated quality and content of motivation. Three studies conducted by Nordbø and Coworkers demonstrated that a treatment independent willingness to recover is a fundamental requirement to readiness to change [17,20,52] and another work showed that patients’ willingness to change, as expressed at the beginning of treatment, is a relevant prognostic factor at six-month follow-up [47]. In line with these findings, an improved motivation to change during therapy represents a crucial factor in overcoming relapses [36]. All these studies highlight how patients’ attitudes towards illness should be accounted for while defining motivation to change [20] and investigated at the beginning of treatment [51].

It is noteworthy that those motivations verbally expressed by patients often do not correspond to an authentic intention to modify their eating disordered behaviors since ED patients can be strongly ambivalent about changing [52]. The ambivalence issue has been confirmed also by another study [42] suggesting the use of the Pros and Cons of Eating Disorders Scale as useful tool to evaluate patients’ perspective of illness. Moreover, a longer duration of illness – index of poor motivation to change – is a negative prognostic factor mostly in AN [39].

Another paper investigated preoccupation with weight and body and found it to be determinant as regards CBT outcome and the chance to overcome treatment resistance [54]. The intensity of such a preoccupation can assume a delusional connotation with repercussions on resistance to treatment by lowering motivation [28] and generating strong ambivalence [30].

Twelve studies highlighted the correlation between patients’ clinical features and their motivation to change. In fact, BN patients are usually more motivated to seek treatment and change than both AN and subthreshold-AN individuals, mostly if chronic [37], and there is general consensus that binge-purging AN individuals show an unfavorable outcome [56]. Another recent study suggested indeed a more positive prognosis for EDNOS individuals; they seemed to achieve a more rapid and stable remission and showed indexes of higher motivation when compared to individuals affected by a full diagnosis [39], providing further support to data already known in literature [33,46]. Those patients with normal body mass index (BMI), showed a more rapid improvement in motivation to change than those with a low BMI [45] and, more in general, baseline BMI was the most significant predictor of outcome in the whole ED diagnostic group [39]. Moreover, poor motivation to change correlated also with laxatives abuse, depression, and body dissatisfaction [35], although some researchers [35] could not find a correlation between clinical severity and poor motivation to treatment, even if more recent studies did not confirm this hypothesis [45]. However, two studies [48,50] indicated that the rapidity of weight restoration is the only significant prognostic factor over the short and medium-term with Lund and Coworkers [48] highlighting indeed how this can indirectly point out an enhanced motivation to treatment. Finally, Schedenbach and Colleagues [57] underscored that the best predictors of treatment outcome are the ability to choose a variety of foods, mostly with high caloric density.

Eleven studies highlighted how motivation to change can quantitatively vary and several papers on AN described different stages of change and their influence on both outcome and resistance. Some authors demonstrated that the extent of clinical improvements can vary also depending on the stage of motivation achieved by patients [61] and that a mismatch between stage of motivation and phase of treatment can enhance resistance to treatment [58]. Other studies correlated motivation-to-change levels to the need and duration of hospitalizations, finding that high motivation correlates with short duration of inpatient treatment and better outcome [49]. Conversely, if the motivation level is low – i.e. pre-contemplation phase according to Prochaska’s model [92] – the need for hospitalizations resulted to be higher [32].

Motivation to change can be improved by sharing treatment plans with patients [41] and can be assessed with the Motivational Interview [93], the Readiness and Motivation Interview [43,44], or the Anorexia Nervosa Stages of Change Questionnaire [55]. Some studies showed that the latter is a useful instrument to predict changes in eating symptomatology [60] and outcome since motivation plays a mediator role between them [34].

#### Maintaining factors and treatment outcome (22 studies)

Although early studies discouraged the search for ED-specific maintaining factors highlighting instead the need of long-term treatments [77], some recent papers called into question the need for their identification and reformulation [70].

Fairburn [65] designed a transdiagnostic cognitive-behavioral therapy for EDs, aiming at addressing maintaining factors; he individuated as main element a scheme of dysfunctional self-evaluation by which patients attribute exaggerated relevance to eating, body shape, and weight. He considered both ED-specific factors (i.e. thoughts about eating, weight, body shape, hyperactivity) and non ED-specific factors like low self-esteem, interpersonal problems, emotional intolerance, and perfectionism. The latter interact with both individual’s specific psychology and other maintaining factors [65].

Perfectionism has been considered also by other authors [78], demonstrating that some of its aspects could represent a transitional status associated to pathology and are no longer present in recovered AN individuals.

In addition, more authors individuated in body image an outcome predictor of hospitalization even more reliable than interpersonal problems and general psychopathology pointing out indeed how body perception instead of body dissatisfaction can be an indicator of treatment progression [63].

Other psychopathological factors with prognostic value were: inadequacy, high asceticism and maturity fear, impulsivity, and sexual problems [18,66-68]. As regards intra-psychic elements instead, one study individuated as ED maintaining factors poor problem solving and relational skills [69].

Five studies considered personality traits as ED maintaining factors. Fassino and Coworkers [66-68] pointed out how low novelty-seeking and high harm-avoidance – along with other psychopathological aspects – represent predictors of poor outcome in an ED multimodal treatment.

Other studies [76] demonstrated instead that narcissistic personality traits were related to strong resistance to weight gain in treated AN individuals and that depressive and psychotic traits entailed better or poorer prognosis, respectively. Another paper [73] with a 9 and 14-year follow-up found that Axis I and II psychopathology could predict both poor outcome and numerous hospitalizations in the ED population. A couple of studies [56,64] confirmed the negative role of general psychopathology on AN prognosis at the 12-year follow-up, whilst another paper [39] found it significant mostly about BN.

Further authors [79] underscored indeed how avoidant personality traits, coupled with a history of sexual abuse, can play a negative role on long-term prognosis after hospitalization. Assuming a categorical approach to study personality, Helverskov and Coworkers [72] acknowledged the presence of a personality disorder as a negative prognostic factor shared by all EDs.

Castro and Colleagues [62] investigated the prognostic meaning of parental bonding on the outcome of short-term therapy in AN. Even highlighting how parental bonding was not particularly different from healthy controls, the authors underscored that parental hyper control as well as having a rejecting father entail are both elements that strongly impact treatments, in addition to ED psychopathology. Recently, some authors [71] supported the role of caregivers’ expressed emotions and reinforcing behaviors as interpersonal maintaining factors. In their work it has been indicated that reducing caregivers’ distress leads to improving patients’ functioning and eating pathology.

Schmidt and Treasure [75] considered as maintaining factors both intrapersonal and interpersonal factors, placing only scarce emphasis on biological elements and body weight. Also Treasure and Coworkers [94] analyzed the interpersonal maintaining factors of EDs pointing out that over protection, coercive treatments, and isolation could be iatrogenic factors.

Finally, two papers investigated psychosocial factors as the lack of a partner, poor family support, and unemployment as relevant predictors of poor outcome at 21 [74] and 12-year follow-up [64]. Another study [39] showed how the scarcity of friends is a negative prognostic factor in EDNOS patients.

#### Therapeutic relationship, countertransference and management of treatment resistance (18 studies)

The studies considered in this thematic area – deepening poor compliance and scarce motivation to treatments – indirectly suggested different models to treat resistant patients.

Carter and Colleagues [36] highlighted how improving and maintaining motivation to treatment during therapy can show a relevant impact on the long-run. Accordingly, it has been shown the need to specifically address motivation as much as social relationships and body image with tailored interventions to obtain an adequate weight restoration, even with acute AN patients [47].

A couple of studies illustrated rehabilitation – with a focus on psychosocial interventions [87] or supportive therapy [86] - constantly advocating the need for tailored-to-person treatments.

Five studies suggested interventions focused more directly on overcoming poor compliance to treatments with an approach aiming at improving motivation to change and treatment. Already Vitousek and Colleagues years ago [89] underscored the importance of improving motivation to change. With their paper, they suggested some cognitive-behavioral strategies that can be applied also to other theoretical models to enhance emotively and cognitively the therapeutic alliance to overcome resistance. In fact, the authors highly recommend to emotionally validate patients by accepting their difficulties and by speaking their language, adopting a Socratic style in the exploration of both ambivalence and resistance to treatment.

Another effective intervention was the Motivational Interview, an approach based on the Socratic method, emphasizing patients’ autonomy and discouraging direct persuasion [84]. Geller and Coworkers [82] demonstrated the effectiveness of Readiness and Motivation Therapy to lower ambivalence and improve change; also Motivational Enhanced Therapy was found to be effective to achieve this goal, even if in a less structured way [80].

A theoretical paper [83] highlighted that clinical interventions should sometimes indulge patients’ resistances; the discharge of poorly motivated patients can be necessary to maintain a therapeutic milieu focused on recovery and to avoid poor compliance [83,89].

Finally, other studies suggested interventions to overcome scarce compliance not directly focusing on patients but rather on therapists, highlighting the need to handle correctly their countertransference since it could play a negative role on treatment [59,85]. Countertransference can be determined by both patients’ [95] and therapists’ features [53,90] and supervision of therapist’s emotions is highly recommended [81].

Patients highly value psychotherapy and therapeutic relationship considering them as useful elements in treatment [69,88]. Accordingly, it has been suggested to work with a particular focus on the patient-therapist interaction and on shared choices [41]. Concluding, a comment article proposed indeed to address the impairment in mentalization skills of ED patients within the therapeutic relationship [24].

## Discussion

The aim of the present paper was to provide a clinical overview of the available literature on resistance to treatments in AN. It is indeed a well-known phenomenon theorized decades ago [89,96,97] that represents a heavy burden for all clinicians [98]. We propose here a scientifically informed discussion since a systematic review of the literature has been hampered by a huge variety of methodologies used in the available studies leading to a lack of comparable data. Therefore, a systematic review of the literature to date would lead only to scarcely powered findings. As a further limitation, it should be born in mind that the biopsychosocial pathogenesis of AN would require to study several sources of knowledge. We will focus on psychosocial factors and emotive and relational impairments, omitting the biological aspects like the effects of starvation on brain functions.

But why is there a dearth of studies on resistance? This apparent disinterest seems to be due to several reasons. Firstly, it is likely that many authors conceive resistance to treatment as a whole with the illness. According to this perspective, the investigation of treatment outcomes in AN would correspond to evaluating resistance too [99]. Secondly, resistance to treatment is a widespread phenomenon. Hence, some of the papers we included in this overview [14,54,100] pointed out how treatment-resistance in the AN field can be easy to notice but difficult to understand. In fact, it is a complex phenomenon that involves vulnerability and maintaining aspects with intertwined biological, psychopathological, and social features [101] that cannot be easily disentangled. Thirdly, in the last years, research focused more on resistance-related biologic aspects [102] rather than on clinical and relational ones [98]. Also psychodynamic psychotherapies have been even less studied although the assessment of resistance represents the core of the psychoanalytic method [103] since resistance can be enhanced by the relationship between patient and therapist. Finally, we could call into question clinicians' poor motivation to study their frustrated therapeutic attempts [94]. Whatever the reason, it may be a mistake not to carefully consider this phenomenon since it is a hallmark of psychiatry [104]. More in detail, since psychotherapy is an effective therapeutic instrument in the ED field, a better understanding of both resistance and strategies to address it should be carefully considered in treatments [2]. From the research questions underpinning this article we sorted our results into four core areas with regard to AN treatment.

### Denial versus insight of illness

This is the most immediate and straightforward correlate of resistance to treatments in AN and it was included even in DSM-IV-TR diagnostic criteria [91]. Denial of illness is defined as the refusal to acknowledge and accept one’s own illness and it refers not only to psychodynamic therapy and defense mechanisms [2] but also to a wider definition of disadaptive coping [105]. Denial of illness is an intrinsic factor of the first phases of AN [91] and it can last years [86], given the ego-syntonic nature of the anorectic disorder [106]. Accordingly, the Academy of EDs has clearly stated that EDs are severe mental illnesses that require a wide and multifaceted health care alert as like as other major psychiatric pathologies including bipolar disorder, schizophrenia, obsessive-compulsive disorder or depression [3].

We included 7 studies converging on the fact that a large number of AN patients deny their disorder [25-31]. This phenomenon can be particularly clear for those patients who need acute hospitalizations: a recent study conducted on a sample of 108 AN inpatients showed that the vast majority of them (63%) deny their illness [107] with 20-30% of cases revealing a symptom-related psychotic status [28,30]. These features may be so widespread because many patients tend to carry out a deliberate denial [27] or minimization [25] of their illness trying to justify – with different degrees of awareness - treatment refusal. In this regard, the data in literature are debated and do not clarify whether denial of illness is a psychosis-like symptom or rather a rigid and disadaptive defense mechanism helping patients to protect themselves by anxiety and depression [26,31] and to avoid treatments [89] or negative emotions [22,23]. It is likely that both these aspects are true; nevertheless, hypothesizing denial as a defense mechanism could be more in line with its time consistency [86], independently of fluctuations in ED symptomatology [28,29].

Data are also controversial about the extent to what denial can impact prognosis since this element seems to be scarcely relevant as regards adolescents [25]. One paper highlighted a linear correlation between insight and duration of illness [31], although this finding has not been replicated [28]. At now, it is not possible to distinguish whether denial is already expressed at the onset of illness or it is enhanced with time and potentially by inappropriate treatments. However, it should be considered as a central issue to be addressed in treatments rather than a prognostic element [29].

The improvement of insight of illness could be an index of a good therapeutic alliance [108]. In fact, the therapeutic relationship is now indeed considered as the most effective instrument to contrast denial of illness [75,109].

In sum, denial of illness, a shared factor with other severe mental disorders [110], was not found to be a predictor of resistance to treatment but rather to correlate with the phenomenon of treatment resistance.

### Motivation to change

Papers on motivation to change in AN allowed a better understanding of denial of illness although one main difficulty encountered in this field of research is to understand patients’ authentic degree of motivation to recover since there is often a difference between what patients do and say [100].

Studies on motivation to change (33 included in this clinical overview) suggested ways to measure this construct [17,20,28,30,32,34-52,55-57,60],[61] and interventions to enhance it [33,58-60]. A lower motivation to change was found to correlate with lower BMI [45] - and in general a more severe eating and general psychopathology [35,39,54]-full diagnosis, purging behaviors [33,45,46,56], lower compliance to dietary recommendations and slow weight gain [48,50,57], and worse quality of life [51]. These data are in line with Kaye’s hypothesis of a vicious cycle of symptoms as maintaining factors in EDs [102].

From the available body of literature emerged a correlation between motivation and psychopathology, even more peculiar in AN than BN [37].

In spite of the number of conducted researches, to date it is still controversial whether motivational interventions can be a main road to improve resistance to treatment or not. There is a significant correlation between willingness to recover and good motivation to change [36], but this association is variable and scarcely supported by other studies [32,34,49,60,61]. Moreover, willingness to recover [52,100,111] or the ability to recognize the negative effects of illness [37] can be confused with motivation to change and this misinterpretation could lead not experienced therapists to enhance resistance through its underestimation. In fact, some studies highlight the need to train therapists to notice, understand and value those emotions that usually underpin AN [97,108,112].

The role of neuropsychological aspects – promising and growing research area [113] – is even less studied and the data in literature are contrasting [38,40]: in fact, cognitive rigidity and impaired decision making – stable traits also in recovered individuals - can only partially represent a hindrance to treatments.

Although several lines of evidence exist in support of motivational interventions [58-61], a recent review conducted by Waller [100] questions their effectiveness since the available studies are frequently biased by methodological flaws. In fact, motivational interventions are not stand-alone treatments; therefore, the psychotherapies (i.e. CBT) that they usually support could be responsible for the real effectiveness of these interventions. Hence, motivational interventions – as to date have been described in literature – can only scarcely impact motivation and failed to significantly improve outcomes with the only exception represented by Binge Eating Disorder patients [100]. Other findings did not provide support for the effectiveness of such interventions with long-standing patients [83].

Some hypotheses could be raised to bridge the gap as regards motivation to change: a) too much emphasis has been placed on words instead of facts [100] without considering patients’ peculiar strive to please [97] and need for approval [108]; b) motivation has been conceived as a too linear concept whilst patients cannot switch directly from one stage to another; c) the assessment of motivational stages is not adequately considered during treatment planning: often motivational stage and phase of therapy do not match, having resistance to treatments enhanced as a result [51,58]; d) motivational models may be too simple and may not to consider patients’ ambivalence in an proper manner [70,114,115]; e) an excessive use of verbal persuasion is usually made at the expense of patient autonomy [100,116].

Therefore, only a few studies tried to investigate the process of recovery in AN and to conceptualize more in detail ambivalence to change and its implications [17].

#### Ambivalence and meaning of illness

In this sense, some papers [17,20,43-45,70] highlighted the relevance of both motivation and pervading ambivalence that paralyzing patients while making their decisions. Some authors pointed out the existence of a sort of “anorexic voice”, an inner entity disapproving patients and being sometimes overwhelming in respect to their sense of the self [117,118]. This voice is even more pervading when the illness gets worse, contributing to hamper treatments. It has been suggested that the link between patients and this AN voice could play a role in unravelling the issue of ambivalent attitudes towards change, typical of BN individuals too [70]. Thus AN and BN patients would constantly struggle between facing resistance to treatment or valuing it.

From a therapeutic perspective, it could be useful to become familiar with the adaptive function of AN and its pros and cons, as experienced by the patients [70,115]. Therefore, it becomes possible not only to perform a cognitive restructuring, but also to mitigate their distress [24] with two aims: a) to use the empathic approach to understand patients’ inner world [89] and to dialogue on both diagnosis [119] and recovery meanings [17]; b) to move the focus of the intervention from pathological beliefs to therapeutic relationship [120].

One way to start addressing resistance to treatment in ED patients is trying to understand the subjective meanings of the illness [75] and patients’ environment [121]. We found 5 articles that consider in detail patients’ meanings of the disorder and they all agree on considering the “positive” functions of AN as enhancing treatment resistance [20]. In fact, the ED can be a way to feel safe, avoid threatening emotions, communicate with others, and feel strong, special and in control [21].

Considering patients’ perspectives for treatments could have several positive implications. First, patients feel themselves as empathically understood [83,108,122,123]; second, it becomes possible to deepen and personalize the comprehension of those feelings that underpin the ED [97] since they can be very different between patients [20]. Third, it is possible to introduce mind and cognitions in therapy – in addition to eating behaviors and body distortions - to avoid resistance and relapses [124]: in fact, if decades ago therapies tended to be excessively focused on family and intra-psychical aspects, now we could incur an opposite risk. Weight restoration should be the starting point of a treatment instead of its main goal. The eating pathology should be considered more as a disturbance of corporeality and as an impairment of embodiment [125] rather than a neurological body image distortion [126]. Fourth, talking about what patients think and feel about the meaning of their illness could provide a therapeutic framework, enabling them to experience their autonomy [120] and supporting an empathic relationship. In fact, it is not possible to face the illness without “being with” the patient [16].

However, the attention to the meanings that patients confer to their disorder is a necessary but not sufficient condition to understand the phenomenon of resistance to treatment. In fact, the therapist risks to become emotionally indulgent with iatrogenic and resistance-enhancing results [94]. It is crucial for the therapist to achieve a “firm empathy” [127] because an empathic understanding of the patient is not enough; firm boundaries are of vital importance to counterbalance empathy in the therapeutic relationship. Such elements are not only cognitive but have also a relational meaning and function. Therapists need to address patients’ need for boundaries, even if not verbally expressed. The treatment, with its implicit relational instruments [128], enables patients to perceive their therapists as both holding [97,129] and handling [89,129] their self-harming attempts. This could be the result of the struggle for control to achieve a sense of identity that Bruch pointed out decades ago highlighting also that for many AN individuals “the experience of being listened to appeared to be of utmost importance … instead of having their feelings and the meaning of their communication interpreted” [97]. This balance between the two elements – firmness and empathy - can promote changes in patient’s personality and coping through interiorization. Certain pathological behaviors should not be allowed or clearly prohibited. At the same time, it is useful to understand patients’ resistances, objections and even their need to feel alone, refused, and poorly understood.

Therapists may also incur the risk to consider only patients’ conscious meanings of illness and to underestimate those unconscious, deeper, and even more distressing. In this sense, conscious meaning can over the long-term hide useful elements in treatment and therefore contributing to treatment-resistance [75].

In sum, the clinical effort performed on the meanings of illness – either conscious or not – could help overcome resistance to treatment. The understanding of patients’ inner world and attitudes towards the illness can become an operational tool to address the core of treatment-resistance within the therapeutic relationship.

### Maintaining factors and treatment outcome

The complex available models to address resistance to treatment are mainly focused on AN maintaining factors and with this overview we found 22 articles investigating this research area showing interesting and well-organized models [11,18,39,56,62-79]. The shared core is the attention to both “symptom treatment” eventually entailing a vicious cycle [5,18,66-68] and negative effects of starvation on brain [130]. Also body image distortions should be addressed in detail [63,65] and body dissatisfaction has been found to correlate with certain styles of attachment [108]. There is robust evidence showing that mental health cannot be reached without recovery of weight, body perception, obsessive thinking on food and body and without regaining a good quality of life.

Moreover, the different models consider personality and interpersonal aspects as premorbid or maintaining factors. Several factors have been called into question as enhancing treatment resistance: 1) low self-esteem and 2) mood intolerance [65]; 3) perfectionism [65,78]; 4) body experiences [17,125]; 5) general psychopathology [39,56,64]; 6) personality [66-68,72,73,76,79]; 7) interpersonal relationships [65,75]; 8) cognitive inflexibility and 9) avoidance of experience and emotions [29,131,132]; 10) care givers’ expressed emotion [14,62,71,75]; 11) poor problem solving abilities [69];12) scarce social support [64,74]; and 13) reduced relational abilities [39,69,133] (for a review on widely used treatment models see: Hay and colleagues [134]). Evidence on a relationship between dropout, treatment response and both character and temperament [14,67,135] encourages to focus on considering in detail patients’ personality.

The extension of research to areas including not only eating symptomatology seems timely and promising: clinicians will probably obtain more instruments to understand their patients, individualize treatments, and handle resistance. However, to date there are no findings supporting such a more articulate approach to AN as regards treatment response. This model can be indeed too detailed for those patients with a less severe psychopathology [136] and RCT are not currently available [109]. Rather- although further studies are needed - an RCT [137] did not show any significant effect in increasing treatment response.

For those patients with an enduring AN and consolidated maintaining factors, supportive or rehabilitative therapies have been suggested [58,87,138,139] since it is unlikely that certain severe patients will respond to treatments being aware of their resistance. Although these approaches are interesting and potentially useful, to the best of our knowledge there is still no clear consensus in literature on the criteria used to define the chronic course in the ED field [87], making even more problematic to group those patients who would benefit from such supportive treatments.

### Therapeutic relationship, countertransference, and management of treatment resistance

Although further research is needed, the aforementioned studies allowed a better understanding of the AN pathogenesis although they were not effective enough in improving prognosis. Sometimes we cannot see woods for trees and - focusing too much on specific aspects - we could miss the overall emotive exchanges constituting the therapeutic relationship [24,97] and its complex patterns of interaction [53].

We retrieved 18 studies investigating the role of the therapeutic relationship on resistance to treatments in AN. It is of interest that these works on one hand refer to decades ago [89], whilst on the other have been only recently conducted [47]. These elements show well the gap in literature that now some authors are trying to fill although both Bruch [97] and Garner [140] underscored the issue of the iatrogenic effect of those therapists who are not able to manage their own emotions. All these papers agree on the importance of emotive aspects within the therapeutic relationship, in treating treatment-resistant AN people. Patients themselves – when describing their recovery process – individuate psychotherapy and relationships as fundamental tools to overcome resistance [88]. In particular, psychotherapy has been described as a continuous and significant experience to achieve self-validation [69]. Such opinions match some authors’ suggestions highlighting the relevance of sharing treatment plans with patients [41].

In this regard, resistance to treatments should be considered within the therapist-patient interaction [84] involving on one hand patients’ and illness features [116] and on the other therapist-related factors [98] and their interaction [90]. In particular, the avoidant [66-68,79,141] and narcissistic [76] personality traits of AN patients, in addition to their disadaptive management of anger [142,143], make the therapeutic alliance difficult, sometimes enhancing the illness and patient’s relational isolation.

In fact, EDs – assuming an overarching psychodynamic perspective – are essentially disorders of the development of the self and personality, as Bruch [97] originally conceived and as Skårderud [24] and Stanghellini and Coworkers [125] have recently suggested, placing emphasis also on insecure attachment [108,144] and mentalization impairments [120,145].

In particular, AN patients’ deep emotions are characterized by fear, emptiness, anger, and profound demoralization [116,142,143,146]. The illness is a desperate and self-harming attempt to control distress, on one hand avoiding emotions and on the other hand expressing them in an exaggerated way or developing an exasperated perfectionism [95,147]. In general, emotion avoidance and dysregulation are mostly related to treatment-resistance [95].

Such emotions reverberate in line with therapists’ ones, mainly if they are young [98] or lacking supervision [81]. Particularly frustration and anger, but also despair, excessive worry, boredom, and feeling of being manipulated [81,85,98] are common when investigating therapists’ countertransference features. Resistance to treatments itself is thought to be the most challenging aspect of the AN treatment, according to studies conducted on clinicians’ perspectives [98]. In this regard, treatment-resistance could be linked to a contagious fear of aggressiveness and despair that could involve the therapist too.

Treatments could be influenced by an overemphasis on cognitive [24] or explicit communication factors, whilst the issue could be related to the avoidance of an emotive confrontation on symptomatology and real-life experiences or, more simply, to the lack of an authentic relationship between patient and therapist [16]. An effective therapy to overcome resistance to treatments could be indeed a cognitive-analytic therapy as Dare and Coworkers [148] suggested and tested with RCT.

Psychotherapy – as well as those therapies focused on AN symptomatology – should then help patients to achieve a multi-dimensional understanding of themselves and to manage their feelings and relationships, gradually reshaping the adaptive function of the illness. This psychotherapeutic model was only sporadically tested in literature [68,135] and - although it may look outdated - it could be proposed again in the light of the studies on psychotherapy currently available.

In fact, psychodynamic psychotherapy is currently regaining its role in psychiatry [149], mostly as integrative discipline and science of intimacy useful to achieve a developmental psychopathology and overcoming its traditional concept of science of interpretation [128]. Neurosciences significantly highlighted how relational our mind is [150] and that human beings are wired to be social [151]. Initial findings showed that shared emotions can synchronize brain activity [152]: from a meta-analytic studies of dynamic psychotherapies we now know that they are effective in several mental disorders [153] and that the more they consider affective and emotional aspects the more effective they are [154].

In psychodynamic psychotherapies, two aspects have been considered as key-elements: a) a secure, sensitive, and interactive therapeutic alliance; and b) encouraging patients to experience the previously avoided threatening feelings [155]. It is the time to (re)introduce these therapeutic processes in the study of AN treatments. Moreover, the concept of resistance to treatments arose from psychoanalysis, as recently remarked [156], on the basis of Freud’s statements [157] clarifying that resistance is intertwined with treatment and that it represents a compromise between the strengths related to recovery and those opposing to it.

AN patients ask their clinicians acceptance, intensity, challenge and mostly competence [53], confronting their knowledge, but even more their relational skills. Being able to provide an empathic understanding is fundamental to train the patients to recognize themselves, and restrain their distress with a good balance of implicit and explicit messages in the here and now of the therapeutic relationship through transference and countertransference [158].

Psychotherapy cannot be manualized enough to avoid the unpredictability of the relationship [128]. To improve the quality of the therapeutic relationship the therapist needs to be authentic, implicit and empathic [128,159]. If the attunement of the therapeutic relationship turns so profound and intense to become embodied simulation [160], also through mirror neurons [161], therapist and patient can start to share not only the distress but also the ability to limit it. Studies on personality and EDs confirm how often emotional coping can be impaired [162]. Therapist’s coping skills can be a useful model to enhance the development of patient’s coping. In fact, affected individuals can implicitly feel and consider the change, starting to overcome their resistance and fear, as like as therapists handle their fears of being too frustrating or too sympathetic with their patients [11].

In the therapeutic relationship, AN patients can experience new theories of others’ mind [163] and more adaptive forms of reflective self-functioning [164] and through this integration of psychic realities they will be again more aware of their own body [24].

Unfortunately, to date there is still little in the way of addressing the treatment-resistance issue in AN. The studies on the importance of therapeutic relationship in facilitating emotive experiences are still sparse or provide only pilot data [143,165]. Although an RCT has been designed in this regard [166], further studies are still warranted to bridge this gap.

## Conclusions

Resistance to treatment and reluctance to recovery represent key-problems in the treatment of individuals affected by AN [13,17]. In fact, affected patients often show poor motivation to treatment entailing high levels of dropout [14,100] and negative outcomes with the illness becoming often chronic and eventually mortal [167].

The present work individuated 71 studies that specifically addressed treatment-resistance but - although many factors are possibly involved – a dearth of evidence-based findings in this area emerged.

To date are available more theoretical models rather than evidence-based studies on the most effective way to manage treatment resistance. Future research is warranted to fill these gaps and to pave the way for a better understanding of EDs and their treatments, although a more clear description of resistant patients emerged from the available findings.

As Strober wrote [139], EDs do not randomly affect individuals, but a certain temperament coupled with immature, avoidant and perfectionistic personality traits make people more vulnerable and prone to the ED onset [162,168]. Therefore, to earn a resistant patient’s trust it is first necessary to recognize the defensive nature of the ED symptoms being also aware of their adaptive function to achieve the mitigation of a profound distress [24]. Hidden by the hyper control of body and food, the main elements are demoralization [141], anger [142,169], low self-esteem, and a great “hunger” of approval and reassurance. AN patients are highly ambivalent about relationships; a disadaptive style of attachment poses the bases for the development of relationships characterized by dependence and fear of others’ opinions. Coping abilities are impaired: emotions are ignored and avoided through alexithymia, or are uncontrollable and destructive generating acting-out. In a specular way, the therapist incurs the risk of becoming alexithymic [170], or angry and frustrated [98].

Several aspects need to be considered to avoid these mechanisms: expertize, firmness, awareness of maintaining factors, and mostly a “relational knowledge”; according to Roland Barthes: “no power, a little knowledge, a little wisdom and as much flavor as possible” [171]. There is no possible treatment of symptoms and cognitive distortions without attunement [128,160]. Who can motivate the treatment-resistant patient to activate a “voluntary suspension of distrust” [172] to overcome such highly valuable symptoms? Only those therapists who can empathically understand those feeling of uselessness, loneliness, and death that patients often experience and highly value.

Treating resistant patients is a long and winding road entailing inevitable multiple problems in the therapeutic alliance. A secure and firm relationship and the avoidance of premature interpretations and arrogant approaches are both effective elements in fostering a positive therapeutic relationship; it is also fundamental indeed to accept and respect patients’ thoughts and wishes [97].

Patients should be provided with a chance to express themselves – even with difficulty – since their most authentic parts are likely to reemerge from the eating obsessions and regain meaning. Only then the psychotherapeutic interventions will not be a priori refused but implemented and sustained by the patients who will be free to regain confidence with their own personality and experiences, also related to food and body. Patients’ profound demoralization [107] will be then mitigated with an encouraging relational strategy [173-175] aimed at the development of the self [97].

Is this approach possible in this economic climate and with the current strong need for cost-effective interventions? It could be feasible whether public health policies will be able to consider wider investments also because AN-related costs are considerable and –according to the available data - may be even underestimated [176]. It is well-known that an incorrect treatment of these disorders could entail patients’ frustration and increase costs [177]. Other severe and resistant psychiatric disorders could benefit from intensive interventions with economic advantages over the medium-term [178,179]; in fact, costs tend to be higher if a correct psychotherapeutic approach is not provided [180].

The dearth of ED programs may be due to the lack of proper knowledge with sometimes clinicians’ attitudes and stigma influencing negatively the availability of ED services [98].

In the future it will be indeed necessary a strong effort as regards both public health decisions and clinical training to finally improve ED treatments and prognosis [167], mostly because these disorders affect young individuals and are still largely unclear.

This overview shows some limitations. First, the possible biological factors related to resistance have not been considered. Second, resistance could be addressed also by other articles that are not specifically dedicated to this issue and so we could have omitted some papers. Third, this wide heterogeneity made it particularly challenging to systematically and critically review the papers included in the present work. Therefore, they have been clinically and theoretically discussed. Finally, the need to synthesize such complex and wide topics may have left some aspects not fully covered.

Further studies will have to validate the need for personalized treatments – mostly psychotherapies - [16,181] and for interventions tailored to personality dimensions to attenuate resistance and prevent dropouts. This statement is not in line with some studies [182,183] highlighting that ED treatments – including the specialist supportive clinical management - are all equally effective. But these studies show a relevant selection [184] and randomization bias: patients did not receive a tailored treatment but a default intervention that did not consider patients’ peculiar meaning of the disorder. Such research methods can indeed uniform results and potentialities of treatments. The negative emotions that underpin the eating symptomatology [147] should be properly addressed with individualized interventions [148]. A recent RCT [185] demonstrated that focal psychodynamic therapy is an effective treatment for AN. It could represent a promising therapeutic opportunity mostly for those patients who are resistant to treatment since psychodynamic psychotherapy can be highly individualized. Further studies testing the application of this intervention also on resistant patients are needed.

In psychiatry, the therapeutic relationship - crucial also during medication prescription [186] - shows even more importance in psychotherapy [187]: the overcoming of resistance to treatment in AN cannot be possible without a deep patients’ understanding, mostly regarding their profound and unique despair, and without sharing with them a long and difficult therapeutic journey [124]. Patients can finally achieve a more mature personality balancing their deficits of the self with strategies to regain confidence in their body and to favorably give up the eating symptomatology.

## Abbreviations

EDs: Eating disorders; AN: Anorexia nervosa; BN: Bulimia nervosa; EDNOS: Eating disorder not otherwise specified; DSM-IV-TR: Diagnostic and statistical manual of mental disorders, fourth edition, text revised; BMI: Body mass index; RCT: Randomized controlled trial; HC: Healthy controls; CBT: Cognitive behavioural therapy.

## Competing interests

The authors have no financial competing interest to disclosure. As regards non-financial competing interests, since SF is a member of the editorial board of the journal, he specifically asked not to be involved in editorial process of this manuscript.

## Authors’ contributions

SF conceived this work and supervised the manuscript in all phases of its preparation; GAD wrote both introduction and discussion and critically revised the paper; ND conducted the literature search and wrote part of the result section; FA conducted the literature search and wrote part of the result section; CDB revised extensively the paper. All authors read and approved the final manuscript.

## Pre-publication history

The pre-publication history for this paper can be accessed here:

http://www.biomedcentral.com/1471-244X/13/294/prepub
