# Correction: Resistance to treatment and change in anorexia nervosa: a clinical overview

**DOI:** 10.1186/1471-244X-14-62

**Published:** 2014-03-04

**Authors:** Giovanni Abbate-Daga, Federico Amianto, Nadia Delsedime, Carlotta De-Bacco, Secondo Fassino

**Affiliations:** 1 Department of Neuroscience, Eating Disorders Center for Treatment and Research, University of Turin, Turin, Italy

## Text

Due to an error in the publication process, this article [1] was published with an incorrect title. The correct title of the article is ‘Resistance to treatment and change in anorexia nervosa: a clinical overview’.

Readers should be aware that this article may still be indexed under its previous, incorrect title ‘Resistance to treatment in eating disorders: a critical challenge’.

BioMed Central apologize to the authors and readers for the error and any inconvenience caused.

## Pre-publication history

The pre-publication history for this paper can be accessed here:

http://www.biomedcentral.com/1471-244X/14/62/prepub
